# Study on the Dynamic Difference between Single and Mixed Residues of Three Neonicotinoids in *Brassica chinensis* L.

**DOI:** 10.3390/molecules26216495

**Published:** 2021-10-27

**Authors:** Yangyang Lu, Qinxiong Rao, Qicai Zhang, Xing Liu, Wei Song, Shuhui Guan, Shanshan Chen, Weiguo Song

**Affiliations:** 1 Institute for Agri-Food Standards and Testing Technology, Shanghai Academy of Agricultural Science, Shanghai 201403, China; yyanglz@163.com (Y.L.); qinxiongrao@163.com (Q.R.); qicaizhang@126.com (Q.Z.); liuxinglyg@126.com (X.L.); songwei890214@163.com (W.S.); shuhuiguan@163.com (S.G.); cssm100@163.com (S.C.); 2 Shanghai Service Platform of Agro-Products Quality and Safety Evaluation Technology, Shanghai 201403, China

**Keywords:** neonicotinoids, insecticide mixtures, dynamic residues, dissipation, *Brassica chinensis* L.

## Abstract

Multiple insecticides’ residues after the mixed application of several neonicotinoids cause combined pollution and bring new challenges to food safety and pest control during agricultural production. In this study, three neonicotinoid insecticides, namely imidacloprid (IMI), acetamiprid (ACE), and thiamethoxam (TMX), were mixed and evenly sprayed on *Brassica chinensis* L. in the field. Then, the insecticides’ residues were dynamically monitored to determine the differences in their rates of dissipation and final residues after 10 days. The results showed that the dissipation kinetics of neonicotinoids still conformed to the first-order kinetic model for binary or ternary application of neonicotinoid mixtures, with all determination coefficients (R^2^) being above 0.9 and the dissipation half-life (DT_50_) being 2.87–6.74 d. For treatment groups with five times the recommended dosages (IMI 300 g·hm^−^^2^, ACE 900 g·hm^−^^2^, and TMX 600 g·hm^−^^2^), mixed insecticides had a slower dissipation rate, and the DT_50_ values of mixtures were longer than those of single insecticides. Moreover, the final insecticide residues with mixed application were higher than those of single compounds at 10 d after spraying. Thus, mixed applications of neonicotinoids may increase food safety risks as they increase the final insecticide residues in *Brassica chinensis* L., and care should therefore be taken when considering the combined use of such compounds.

## 1. Introduction

Neonicotinoids have come to be the most widely used insecticides around the world since their introduction in the 1990s [1]. Due to their systematic properties, neonicotinoids can be absorbed by the roots or leaves and translocated to all tissues as the crop plant grows. Moreover, neonicotinoids are beneficial for the efficient control of aphids, leafhoppers, planthoppers, water weevils, lepidopteran leaf miners, whiteflies, and other pests in the field of agriculture [2,3].

The main neonicotinoid insecticides are imidacloprid (IMI), acetamiprid (ACE), and thiamethoxam (TMX), which are all best-sellers in the global market. However, their residues are frequently found in a variety of foods and environmental samples. Previous investigations demonstrated that neonicotinoids and their metabolites were present in drinking water [4,5,6], vegetables, fruits [7], bovine milk [8], honey [9], and other food crops [10]—in some cases exceeding the maximum residue limits (MRLs) in agro-food samples [11,12]. Three neonicotinoids’ (IMI, ACE, and TMX) residues were detected in all 49 vegetable samples from a market in Beijing [13]. In addition, the coexistence of multiple neonicotinoids’ residues was the other serious issue in these investigated samples. Three neonicotinoids coexisted in raisins, spinach, kale, and strawberries [14]. Of 343 basil samples from the USA, 5% contained IMI, ACE, and TMX simultaneously, and the levels in 7–13 basil samples exceeded their maximum residue limit [15]. Generally, insecticides with multiple active ingredients are selected to prevent various pests in agriculture [16], resulting in residue mixtures of multiple insecticides. It is not certain that behaviors under single insecticide application in agriculture can be used to explain the dissipation and residues of insecticides in mixed application.

Assessing and monitoring residues is a critical step in the proper assessment of human exposure to insecticides through foods [17]. The cumulative risk assessment of some insecticides with the same action mechanism was proven to result in additive, synergistic, or other mixed effects to human health [18]. Many studies showed that multi-chemical mixtures had significant toxic effects, even if the concentrations of individual chemicals were below their no-observed-effect concentrations [19,20]. Compared with single insecticide application, mixtures of IMI, clothianidin, and TMX had directly additive effects [21]. Therefore, although harmful single residues did not exceed the standard, insecticide mixtures resulted in excess residues due to the mixed effects and posed emerging food safety risks to consumers.

Most studies have focused on the combined toxicity effects of insecticide mixtures. However, the interaction between the insecticide mixtures and their environmental behaviors is also important for controlling residue risks. It is necessary to understand the transformation in the agro-food chain and the residue interaction of insecticide mixtures [22,23,24]. Thus, in this study, three neonicotinoids were sprayed on *Brassica chinensis* L. in an open field either alone or in a mixture, and the insecticide residues were dynamically monitored to explore differences. Moreover, the results provide environmental behavior data for cumulative risk assessment and lay the foundation for the scientific and reasonable application of neonicotinoid mixtures.

## 2. Results and Discussion

### 2.1. Neonicotinoid Residues in Aerial Part of Brassica chinensis L.

#### 2.1.1. Method Validation

Seven concentrations (0.2–8 mg·L^−^^1^) of the three neonicotinoids were each dissolved in acetonitrile, and three calibration curves were constructed; the curves’ coefficients of determination (R^2^) were above 0.99. Untreated *Brassica chinensis* L. samples were spiked with 0.1, 10, and 100 mg·kg^−1^ neonicotinoids for the recovery test, replicated five times. The recoveries of IMI, ACE, and TMX were in the range of 97.0–100, 98.4–102, and 94.3–96.1% on average, respectively, and the coefficients of variation were 1.35–5.48, 1.51–6.37, and 1.68–9.02%, respectively. The limit of quantitation (LOQ) of the three neonicotinoids was 0.1 mg·kg^−1^. These results imply that the detection method was suitable for analyzing the residues.

#### 2.1.2. Initial and Final Residues after Mixture Application

**Figure 1:**
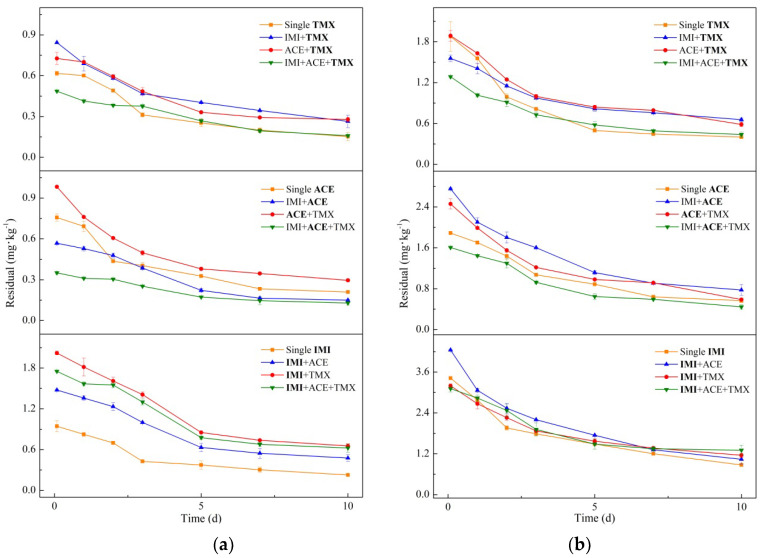
Different recommended doses of three neonicotinoid residues in *Brassica chinensis* L.: (**a**) dose A; (**b**) dose B. The points and error bars represent the mean and standard deviation of replicates, respectively (*n* = 3).

In this study, the initial insecticide residue is defined as the residual insecticide concentration detected after spraying insecticide for 1 to 2 h. It is generally used as a measure of the degree of initial insecticide contamination in/on crops and is closely related to factors such as leaf surface area, method of insecticide application, and dosage. The final residue is the residual insecticide concentration detected at 10 d after spraying insecticide, which provides data support to assess the insecticide risk to human health and the environment. According to the instructions for insecticide application, the recommended dose of IMI is 60 g·hm^−2^, while that of ACE is 180 g·hm^−2^, and that of TMX is 120 g·hm^−2^. In this study, dose A of 5 times and dose B of 20 times the recommend dosage were selected for testing. The dissipation curves of the neonicotinoid residues on *Brassica chinensis* L. with individual and mixed applications are shown in Figure 1.

The results show that at dose A of IMI, the initial and final single residues were 0.944 and 0.227 mg·kg^−1^, respectively, which were lower than the respective mixed application residues of 1.476–2.021 and 0.476–0.654 mg·kg^−1^. At dose B, the final residue in all mixtures was 1.038–1.303 mg·kg^−1^, which was higher than the concentration of 0.872 mg·kg^−1^ from the single applications. Therefore, when applied together with the other neonicotinoids, the IMI residue concentration increased, indicating that IMI probably poses a higher risk to consumers. There was a tendency for a higher initial IMI residue to produce a higher final residue at dose A, but this tendency did not appear at dose B. The highest initial residue of 4.284 mg·kg^−1^ in the IMI + ACE treatment did not produce the highest final residues. Thus, the degree of dissipation may be different when mixed with different insecticides.

The final residue of single ACE was 0.209 mg·kg^−1^ at dose A, and those of mixed IMI + ACE and ACE + TMX were 0.150 and 0.296 mg·kg^−1^, respectively. Single application of ACE at dose B resulted in a residue concentration of 0.567 mg·kg^−1^, while mixed applications resulted in final residues of 0.776 and 0.588 mg·kg^−1^. When applied mixed with TMX, ACE had more residue; thus, TMX had a residue-enhancing effect on ACE. The initial and final residues with ternary application were the lowest compared with single and binary applications, reaching 0.351 and 0.129 mg·kg^−1^ at dose A and 1.607 and 0.444 mg·kg^−1^ at dose B, respectively. Therefore, two neonicotinoids used together may increase ACE residues, but the use of three may decrease them.

The initial residues of single TMX were 0.617 mg·kg^−1^ at dose A, 1.877 mg·kg^−1^ at dose B, and 0.844–1.889 mg·kg^−1^ in the two neonicotinoid mixed applications. The final residues of the two mixtures were 0.264–0.656 mg·kg^−1^, which were higher than the single application residues of 0.152 and 0.401 mg·kg^−1^. When the three neonicotinoids were mixed, the initial TMX residues were 0.486 mg·kg^−1^ at dose A and 1.287 mg·kg^−1^ at dose B, which were lower than the values from the single application, whereas the final residues of the mixture were 0.159 mg·kg^−1^ with dose A and 0.438 mg·kg^−1^ with dose B, which were slightly higher than those from the single application.

These results show that most of the final residues were increased after the mixed applications compared with the single applications. The initial residues were also influenced by application with other insecticides, and most mixtures’ initial residues exceeded the single initial residue concentrations. At present, the efficacy and safety of an insecticide is usually justified by itself, without considering the interactions between multiple insecticides. It was considered that the dissipation of insecticides in tomatoes was not only affected by the climatic conditions, type of application, plant species, and growth dilution factor but also by co-application of the insecticides [25]. It was reported that the dissipation of insecticide residues was affected by the mixed use of insecticides, which were often used by growers [21]. In this study, interactions on the initial and final neonicotinoid residues in vegetables were proven by field trials. In order to analyze the reasons for the differences between the residues, the dissipation kinetic was further analyzed to calculate the dissipation rate and half-life of the neonicotinoid insecticides.

### 2.2. Dissipation Dynamic Analysis of Neonicotinoid Insecticides’ Residues

#### 2.2.1. Data Analysis

The residual neonicotinoid concentrations were analyzed with the first-order kinetic equation to explore the degradation speed of the active substances of the three neonicotinoids in *Brassica chinensis* L.:

(1) $$C_{t}=C_{0}{\;e}^{-\mathrm{kt}}$$

where C_t_ is the residual neonicotinoid concentration (mg·kg^−1^) at time t (d) after application, C_0_ is the initial neonicotinoid concentration (mg·kg^−1^), e is the base of the natural logarithm, and k is the neonicotinoid degradation rate constant (d^−1^). The corresponding degradation half-life (DT_50_) can be calculated as follows [26,27]:

(2) $${\mathrm{DT}}_{50}=\ln2/k$$

where DT_50_ is the neonicotinoid degradation half-life (d), which is used for all neonicotinoids with significant models (R^2^ > 0.5). The model parameters of C_0_ and k were obtained by fitting the curves, which were drawn from all the experimental residue values of each neonicotinoid in *Brassica chinensis* L. (C_t_) and corresponding times after neonicotinoid application (t). All statistical analyses were carried out on SPSS Statistics 17.0 (IBM Co., Armonk, NY, USA), Origin 8.5 software (OriginLab Co., Northampton, MA, USA), and MATLAB R2009a software (MathWorks Co., Natick, MA, USA).

The dynamic model was used to analyze the residual data. According to previous studies [28,29,30], the first-order kinetic model is the most suitable model. As shown in Table 1, the neonicotinoids applied individually or in mixtures were all fitted into the first-order kinetic model, and the R^2^ values ranged from 0.903 to 0.973. A one-way analysis of variance (ANOVA) was performed to examine statistically significant differences. A probability level of *p* < 0.05 was considered significant. Therefore, whether the three neonicotinoids were applied alone or in a mixture, the insecticide residues still conformed to the first-order kinetic dissipation law. However, there must be mutual effects between the neonicotinoids when coexisting in the crops.

#### 2.2.2. Interaction Effect of Neonicotinoid Mixture

The k-value and DT_50_ value can be used to characterize the dissipation rate of insecticides; the higher the value of k is, the lower the DT_50_ value is. The DT_50_ value of IMI alone at dose A was 3.88 d and at dose B was 4.36 d. With mixed IMI and ACE, the DT_50_ value of IMI was 1.06 d at dose A—higher than that under the single application—and 0.18 d at dose B—lower than under the single application. The results showed that mixed application with low concentration could slow down IMI dissipation. The DT_50_ value of mixed IMI + TMX was 4.96 d and that of IMI + ACE + TMX was 5.42 d at dose A; the respective values were 5.82 and 5.99 d at dose B, which were higher than under the single application.

At dose A, the DT_50_ values of mixed IMI + ACE, ACE + TMX, and IMI + ACE + TMX were 4.22, 4.45, and 4.48 d, respectively, being higher than the value of 4.10 d with application of ACE alone; thus, mixed application had an inhibitory effect on dissipation. On the contrary, at dose B, the DT_50_ value of single ACE was 4.66 d, which was higher than those of mixed IMI + ACE (4.52 d), ACE + TMX (4.22 d), and IMI + ACE + TMX (4.48 d). Though the dissipation rate might have increased, there were still more residues because the initial concentration was high.

For TMX alone, the DT_50_ value at dose A was 3.97 d; that for the mixture of IMI + TMX was 5.28 d; and ACE + TMX and IMI + ACE + TMX had DT_50_ values of 5.42 and 5.94 d, respectively. The results showed that mixed application exhibited an inhibitory effect on the dissipation rate. Similarly, mixed IMI + TMX (6.74 d), ACE + TMX (5.01 d), and IMI + ACE + TMX (5.27 d) had higher values than single TMX (2.87 d) at dose B. As with the initial residues and final residues, IMI and TMX applied alone showed lower DT_50_ values than with their mixed application.

The above results showed that IMI and ACE coexistence can speed up dissipation at higher concentrations. Mixed IMI and TMX had an inhibitory effect on the dissipation rate of both. At dose B, TMX had an enhancement effect on ACE dissipation, while ACE showed a greater adverse effect on TMX dissipation. There was a mutual promotion of dissipation between ACE and TMX. Compared to single application, ternary application of the neonicotinoids had inhibitory effects on the dissipation rate, which resulted in more insecticide residues.

MATLAB software was used to conduct a regression analysis of the neonicotinoid mixtures. All coefficients of determination (R) from the regression analysis were close to 1.0 (Table 2), which showed that the regression effect had a good result. According to the results, IMI had a greater influence on the final residues of IMI and ACE in the mixture after mixing the two. The mixture of IMI and TMX at dose A had a greater influence on their respective final residues, but dose A of IMI and TMX individually had a greater influence on both. At dose B, TMX had a greater influence on the final residue of the ACE and TMX mixture. The final residue of the three mixtures was more affected by TMX at a high concentration, but ACE had a greater effect on the three mixtures at a low concentration. Therefore, the presence of TMX had the most dominant impact on the dissipation of neonicotinoids in the mixed applications. Thus, the conclusions of the regression analysis are consistent with the results from the DT_50_ values.

Almost all dissipation half-lives of the mixed neonicotinoids were longer than those of the single ones. The results indicate that the fastest rate of dissipation occurred with the single TMX insecticide, and the slowest dissipation rate occurred with the IMI and TMX mixture at dose A. The reason was that the insecticide dissipation rate might be influenced by mixed application. There are many different mixed effects among different neonicotinoids. According to some previous studies, co-application of oxytetracycline inhibited the removal of two fungicides (carbendazim and metalaxyl) in IF systems and resulted in longer half-lives [31]. Jiang et al. found that oxytetracycline might decelerate the dissipation rate of herbicides by affecting soil microorganisms and enzymes [32]. In this study, the dissipation rate was decelerated in most mixtures, which was similar to the results obtained with a copper–sulfadiazine mixture [33] and a procymidone–TMX mixture [29].

Most neonicotinoids undergo metabolic alterations at multiple sites. IMI residues are determined as the parent compound and metabolites as the chloropyridinyl moiety. IMI is hydroxylated in the imidazolidine moiety at either one of the two methylene substituents, which is followed by conjugation or dehydration to form olefin, apparently with little or no ring opening; these unconjugated metabolites are retained [34]. ACE experiences N-demethylation and cleavage of the N-cyanoacetamidine linkage in plants and its residue as the parent compounds is regulated [35]. TMX residues are considered along with those of its principal metabolite clothianidin. TMX is readily transformed to clothianidin by ring methylene hydroxylation in plants [36], whereas clothianidin undergoes N-demethylation [37]. N-demethylation is observed in each case with compound-dependent effects on product potency, and the effect causes emulative interaction to prevent other neonicotinoids from degrading. The toxic effects of insecticides on wildlife and human health are generally evaluated on compounds individually rather than on the interactions of mixtures. Interactions between insecticides are only tested in a limited quantity of mixtures, and therefore, knowledge of the target site is necessary for the safe and effective use of insecticides in agriculture.

## 3. Materials and Methods

### 3.1. Reagents, Chemicals, and Materials

The *Brassica chinensis* L. plants used in these experiments were from farmland in the Zhuanghang experimental base, Shanghai Academy of Agriculture Science. The neonicotinoids included water-dispersible granules of 70% IMI, water-soluble powder of 20% ACE, and water-dispersible granules of 25% TMX, which were from Bayer Crop Science (Hangzhou, China), Noposion (Shenzhen, China), and Guanlong Agrochemical (Hengshui, China), respectively. Standards samples of IMI (purity > 99.5%), ACE (purity > 99%), and TMX (purity > 99%) were obtained from Dr. Ehrenstorfer (Augsburg, Germany).

High-performance liquid chromatography (HPLC)-grade acetonitrile was from ANPEL (Shanghai, China), and analytical-grade sodium chloride was from Lingfeng (Shanghai, China). A 2-milliliter dispersive solid-phase extraction (dSPE) purifier tube containing 150 mg anhydrous magnesium sulfate, 25 mg primary secondary amine (PSA), and 7.5 mg graphitized carbon black (GCB) was obtained from ANPEL (Shanghai, China). Milli-Q quality water was made using an Ultra-pure Water Purifier (Millipore, Burlington, MA, USA) for experiments.

### 3.2. Field Experiment

The field experiment was based on the open land of the Zhuanghang experimental station (China; southwest of Shanghai; 30°53′39″ N, 121°22′12″ E). There were 43 experimental grid cells in a plot, including 1 control group without any insecticide application and 7 test groups of 2 different concentrations of insecticides in 3 replicates; a plot full of *Brassica chinensis* L. was defined as a zone of 688 m^2^ divided into independent and non-interfering grid cells of 16 m^2^ (4 × 4 m). The insecticides were sprayed on the crop using a Knapsack sprayer (compression sprayer with a fan spraying nozzle and metal spray lance; spray pressure was 0.3 MPa) at the vegetable growth stage. Residue monitoring of the three neonicotinoids was carried out as separate experiments. The aboveground part of *Brassica chinensis* L. was collected and analyzed at 2 h, 1 d, 2 d, 3 d, 5 d, 7 d, and 10 d during the cropping season in December. The environmental parameters were recorded during the study period: the average temperature was 6.7 °C, the average relative humidity was 83.2%, the total cumulative rainfall was 69.4 mm, and the wind speed was 7.6 km/h.

### 3.3. Sample Preparation

*Brassica chinensis* L. plants (approximately 1 kg) were randomly collected from the treated plots and taken back to the lab within 1 h. All the samples were washed with tap water, dried with paper, homogenized in a JYL-C022E Food Processer (Joyoung Company, Jinan, China), and stored at −20 °C before analysis. The samples (5 g) were extracted with acetonitrile (10 mL) via vortexing for 30 min in an EOFO-945008 Talboys Multi-tubular Vortex Mixer (Troemner Company, Thorofare, NJ, USA), plus sodium chloride (2 g) by homogenization via vortexing, then centrifuged using a D-37520 Refrigerated Centrifuge (Thermo Company, Waltham, MA, USA) at 4500 rpm for 5 min. An aliquot of 1 mL was drawn from the supernatant and cleaned by dSPE, followed by vortexing for 1 min and centrifugation in a 5424R High-Speed Refrigerated Centrifuge (Eppendorf Company, Hamburg, Germany) at 12,000 rpm for 3 min. The supernatant was filtered through 0.22-micrometer polyvinylidene fluoride membrane filters, diluted to a linear range by acetonitrile, and then analyzed by UPLC. In addition, there was a group of samples without any insecticide treatment as blank controls.

### 3.4. Analysis by UPLC

Ultra-performance liquid chromatography (UPLC) was performed via a Waters Acquity system (Waters, Milford, MA, USA), and separation was performed on a CORTECS-C18 column (2.1 × 100 mm, i.d. 1.6 µm) at 30 °C, with a mobile phase flow rate of 0.4 mL/min. The mobile phase consisted of acetonitrile and ultra-pure water with a linear gradient elution program as follows: initial 5% acetonitrile, 6 min at 30% acetonitrile, 6.5 min at 5% acetonitrile, and equilibration for 1.5 min, giving a total run time of 8 min. The injection volume was 5 μL. Detection was carried out using a photodiode array (PDA) detector at 256 nm. The retention time of IMI, ACE, and TMX was 4.32, 4.83, and 3.35 min, respectively.

## 4. Conclusions

This study explored the dissipation kinetics of IMI, ACE, and TMX in leafy agro-food with single and mixed applications. The dissipation process of these three neonicotinoids fit well with the first-order kinetics model, and the characteristic parameters of the dissipation process were obtained. Compared with single application, the insecticides’ dissipation rates were slower and the associated DT_50_ values were higher with the mixed application, which implies that there was an interaction between the neonicotinoids when used simultaneously. Additionally, there were higher initial and final residues in most of the neonicotinoid mixtures. Therefore, the mixed use of neonicotinoids may increase insecticide residues on vegetables in the field and may cause an environmental and agro-food safety risk. In particular, mixed application of the three neonicotinoids in agro-food should be avoided.

## Figures and Tables

**Table 1:** First-order kinetic modeling for dissipation of three neonicotinoids in *Brassica chinensis* L.

		Dose A				Dose B			
Insecticide	Treatments	Kinetic Equation	k (d^−1^)	R^2^	DT_50_ (d)	Kinetic Equation	k (d^−1^)	R^2^	DT_50_ (d)
IMI	Single IMI	Ct = 0.948e^−0.179t^	0.179	0.937	3.88	Ct = 3.207e^−0.159t^	0.159	0.920	4.36
	IMI + ACE	Ct = 1.529e^−0.140t^	0.140	0.959	4.94	Ct = 3.903e^−0.166t^	0.166	0.932	4.18
	IMI + TMX	Ct = 2.065e^−0.140t^	0.140	0.962	4.96	Ct = 3.014e^−0.119t^	0.119	0.932	5.82
	IMI + ACE + TMX	Ct = 1.814e^−0.128t^	0.128	0.930	5.42	Ct = 3.063e^−0.116t^	0.116	0.909	5.99
ACE	Single ACE	Ct = 0.744e^−0.169t^	0.169	0.909	4.10	Ct = 1.903e^−0.149t^	0.149	0.966	4.66
	IMI + ACE	Ct = 0.606e^−0.164t^	0.164	0.956	4.22	Ct = 2.593e^−0.153t^	0.153	0.952	4.52
	ACE +TMX	Ct = 0.909e^−0.156t^	0.156	0.903	4.45	Ct = 2.335e^−0.164t^	0.164	0.937	4.22
	IMI + ACE + TMX	Ct = 0.357e^−0.119t^	0.119	0.957	5.83	Ct = 1.644e^−0.155t^	0.155	0.958	4.48
TMX	Single TMX	Ct = 0.651e^−0.175t^	0.175	0.936	3.97	Ct = 1.865e^−0.242t^	0.242	0.933	2.87
	IMI + TMX	Ct = 0.795e^−0.131t^	0.131	0.941	5.28	Ct = 1.494e^−0.103t^	0.103	0.914	6.74
	ACE +TMX	Ct = 0.748e^−0.128t^	0.128	0.935	5.42	Ct = 1.798e^−0.138t^	0.138	0.911	5.01
	IMI + ACE + TMX	Ct = 0.486e^−0.117t^	0.117	0.973	5.94	Ct = 1.206e^−0.132t^	0.132	0.930	5.27

**Table 2:** Regression analysis of neonicotinoid mixtures’ residues in *Brassica chinensis* L.

		Dose A		Dose B	
Insecticide	Treatments	Regression Equation	R	Regression Equation	R
IMI	IMI + ACE	y = 0.175 + 1.29 x_1_ + 0.194 x_2_	0.9695	y = −0.076 + 1.047 x_1_ + 0.310 x_2_	0.9907
	IMI + TMX	y = 0.259 − 0.235 x_1_ + 3.11 x_2_	0.9684	y = 0.555 + 0.561 x_1_ + 0.399 x_2_	0.9918
	IMI + ACE + TMX	y = 0.307 − 0.512 x_1_ + 3.31 x_2_ − 0.206 x_3_	0.9537	y = 0.665 − 0.468 x_1_ + 0.868 x_2_ + 1.27 x_3_	0.9988
ACE	IMI + ACE	y = 0.0215 + 0.532 x_1_ + 0.106 x_2_	0.9594	y = 0.0283 + 0.462 x_1_ + 0.565 x_2_	0.9881
	ACE +TMX	y = 0.0561 + 0.866 x_1_ + 0.316 x_2_	0.9789	y = 0.154 + 0.502 x_1_ + 0.684 x_2_	0.9905
	IMI + ACE + TMX	y = 0.0718 + 0.0155 x_1_ + 0.452 x_2_ − 0.0273 x_3_	0.9652	y = 0.0168 − 0.174 x_1_ + 0.176 x_2_ + 0.980 x_3_	0.9936
TMX	IMI + TMX	y = 0.121 + 1.24 x_1_ − 0.741 x_2_	0.9902	y = 0.427 + 0.144 x_1_ + 0.363 x_2_	0.9921
	ACE +TMX	y = 0.114 + 0.123 x_1_ + 0.852 x_2_	0.9880	y = 0.264 + 0.319 x_1_ + 0.535 x_2_	0.9940
	IMI + ACE + TMX	y = 0.0979 − 0.0764 x_1_ + 0.414 x_2_ + 0.261 x_3_	0.9353	y = 0.118 + 0.102 x_1_ + 0.078 x_2_ + 0.334 x_3_	0.9915

## Data Availability

The data presented in this study are available within the article.

## Sample Availability

Samples of the compounds are not available from the authors.

## References

[1] Kazuhiko M., Makoto I., David B.S. (2020). Neonicotinoid Insecticides: Molecular Targets, Resistance, and Toxicity. Annu. Rev. Pharmacol..

[2] Elbert A., Haas M., Springer B., Thielert W., Nauen R. (2008). Applied aspects of neonicotinoid uses in crop protection. Pest. Manag. Sci..

[3] Ge J., Cui K., Yan H., Li Y., Chai Y., Liu X., Cheng J., Yu X. (2017). Uptake and translocation of imidacloprid thiamethoxam and difenoconazole in rice plants. Environ. Pollut..

[4] Seccia S., Fidente P., Barbini D., Morrica P. (2005). Multiresidue determination of nicotinoid insecticide residues in drinking water by liquid chromatography with electrospray ionization mass spectrometry. Anal. Chim. Acta.

[5] Van Dijk T.C., Van Staalduinen M.A., Van der Sluijs J.P. (2013). Macro-invertebrate decline in surface water polluted with imidacloprid. PLoS ONE.

[6] Klarich K.L., Pflug N.C., DeWald E.M., Hladik M.L., Kolpin D.W., Cwiertny D., LeFevre G.H. (2017). Occurrence of neonicotinoid insecticides in finished drinking water and fate during drinking water treatment. Environ. Sci. Technol. Lett..

[7] Xie W., Han C., Qian Y., Ding H., Chen X., Xi J. (2011). Determination of neonicotinoid pesticides residues in agricultural samples by solid-phase extraction combined with liquid chromatography-tandem mass spectrometry. J. Chromatogr. A.

[8] Seccia S., Fidente P., Montesano D., Morrica P. (2008). Determination of neonicotinoid insecticides residues in bovine milk samples by solid-phase extraction clean-up and liquid chromatography with diode-array detection. J. Chromatogr. A.

[9] Mitchell E.A.D., Mulhauser B., Mulot M., Mutabazi A., Glauser G., Aebi A. (2017). Aworldwide survey of neonicotinoids in honey. Science.

[10] Main A.R., Headley J.V., Peru K.M., Michel N.L., Cessna A.J. (2014). Widespread use and frequent detection of neonicotinoid insecticides in wetlands of Canada’s prairie Pothole region. PLoS ONE.

[11] Kapoor U., Srivastava M.K., Srivastava A.K., Patel D.K., Garg V., Srivastava L.P. (2013). Analysis of imidacloprid residues in fruits vegetables cereals fruit juices and baby foods and daily intake estimation in and around Lucknow India. Environ. Toxicol. Chem..

[12] Jallow M.F.A., Awadh D.G., Albaho M.S., Devi V.Y. (2017). Ahmad N Monitoring of pesticide residues in commonly used fruits and vegetables in Kuwait. Int. J. Environ. Res. Public Health.

[13] Tan Y., Zhang Q., Zhao C., Wang X., Li J., Wang D., Zhou Y., Lu X. (2016). Residues of Neonicotinoid Pesticides in Vegetables and Fruit and Health Risk Assessment of Human Exposure via Food Intake. Asian J. Ecotoxicol..

[14] USDA (2018). Pesticide Data Program: Annual Summary Calendar Year. https://www.ams.usda.gov/sites/default/files/media/2018PDPAnnualSummary.pdf.

[15] USDA (2019). Pesticide Data Program: Annual Summary Calendar Year. https://www.ams.usda.gov/sites/default/files/media/2019PDPAnnualSummary.pdf.

[16] Felix F.S., Dajana L., Hannes P., Almut M., Joern K., Andreas E.S., Thomas O.J., Albert B., Oliver P. (2021). Pesticide mixture effects on liver protein abundance in HepaRG cells. Toxicology.

[17] Farouk M., Hussein L.A.E., El-Azab N.F. (2016). Simultaneous determination of three neonicotinoid insecticide residues and their metabolite in cucumbers and soil by quechers clean up and liquid chromatography with diode-array detection. Anal. Methods.

[18] Hernandez A.F., Parron T., Tsatsakis A.M., Requena M., Alarcon R., Lopez-Guarnido O. (2013). Toxic effects of pesticide mixtures at a molecular level: Their relevance to human health. Toxicology.

[19] Faust M., Altenburger R., Backhaus T., Blanck H., Boedeker W., Gramatica P., Hamer V., Scholze M., Vighi M., Grimme L.H. (2003). Joint algal toxicity of 16 dissimilarly acting chemicals is predictable by the concept of independent action. Aquat. Toxicol..

[20] Silva E., Rajapakse N., Kortenkamp A. (2002). Something from “nothing”-eight weak estrogenic chemicals combined at concentrations below NOECs produce significant mixture effects. Environ. Sci. Technol..

[21] Maloney E.M., Liber K., Headley J.V., Peru K.M., Morrissey C.A. (2018). Neonicotinoid insecticide mixtures: Evaluation of laboratory-based toxicity predictions under semi-controlled field conditions. Environ. Pollut..

[22] Backhaus T., Blanck H., Faust M. (2010). Hazard and Risk Assessment of Chemical Mixtures under REACH-State of the Art, Gaps and Options for Improvement. BMC. Evol. Biol..

[23] Backhaus T., Faust M. (2012). Predictive environmental risk assessment of chemical mixtures: A conceptual framework. Environ. Sci. Technol..

[24] Belden J.B., Gilliom R.J., Lydy M.J. (2007). How well can we predict the toxicity of pesticide mixtures to aquatic life?. Integr. Environ. Assess Manag..

[25] Khay S., Choi J.H., Abd El-Aty A.M., Mamun M.I., Park B.J., Goudah A. (2008). Dissipation behavior of lufenuron benzoylphenylurea insecticide in/on Chinese cabbage applied by foliar spraying under greenhouse conditions. Bull. Environ. Contam. Toxicol..

[26] Fantke P., Juraske R. (2013). Variability of pesticide dissipation half-lives in plants. Environ. Sci. Technol..

[27] Fantke P., Gillespie B.W., Juraske R., Jolliet O. (2014). Estimating half-lives for pesticide dissipation from plants. Environ. Sci. Technol..

[28] Paramasivam M., Deepa M., Selvi C., Chandrasekaran S. (2017). Dissipation kinetics of beta-cyfluthrin and imidacloprid in tea and their transfer from processed tea to infusion. Ecotox. Environ. Safe.

[29] Lin S., Han Y., Jiangyuan C., Luo Y., Xu W., Luo H., Pang G. (2019). Revealing the biodiversity and the response of pathogen to a combined use of procymidone and thiamethoxam in tomatoes. Food Chem..

[30] Zhang Y., Hu S., Zhang H., Shen G., Zhang W. (2017). Degradation kinetics and mechanism of sulfadiazine and sulfamethoxazole in an agricultural soil system with manure application. Sci. Total Environ..

[31] Huete-Soto A., Masís-Mora M., Lizano-Fallas V., Chin-Pampillo J.S., Carazo-Rojas E., Rodríguez-Rodríguez C.E. (2017). Simultaneous removal of structurally different pesticides in a biomixture: Detoxification and effect of oxytetracycline. Chemosphere.

[32] Jiang W., Gao J., Cheng Z., Wang P., Zhou Z., Liu D. (2018). The effect of antibiotics on the persistence of herbicides in soil under the combined pollution. Chemosphere.

[33] Xu Y., Yu W., Ma Q., Wang J., Zhou H., Jiang C. (2016). The combined effect of sulfadiazine and copper on soil microbial activity and community structure. Ecotoxicol. Environ. Safe.

[34] Nauen R., Ebbinghaus-Kintscher U., Schmuck R. (2001). Toxicity and nicotinic acetylcholine receptor interaction of imidacloprid and its metabolites in Apis mellifera (Hymenoptera: Apidae). Pest. Manag. Sci..

[35] Roberts T.R., Hutson D.H. (1999). Metabolic Pathways of Agrochemicals.

[36] Nauen R., Ebbinghaus-Kintscher U., Salgado V.L., Kaussmann M. (2003). Thiamethoxam is a neonicotinoid precursor converted to clothianidin in insects and plants. Physiology.

[37] Yokota T., Mikata K., Nagasaki H., Ohta K. (2003). Absorption, tissue distribution, excretion, and metabolism of clothianidin in rats. J. Agric. Food Chem..

